# Identified GNGT1 and NMU as Combined Diagnosis Biomarker of Non-Small-Cell Lung Cancer Utilizing Bioinformatics and Logistic Regression

**DOI:** 10.1155/2021/6696198

**Published:** 2021-01-06

**Authors:** Jia-Jia Zhang, Jiang Hong, Yu-Shui Ma, Yi Shi, Dan-Dan Zhang, Xiao-Li Yang, Cheng-You Jia, Yu-Zhen Yin, Geng-Xi Jiang, Da Fu, Fei Yu

**Affiliations:** ^1^Department of Nuclear Medicine, Shanghai Tenth People's Hospital, Tongji University School of Medicine, Shanghai 200072, China; ^2^Department of Thoracic Surgery, Navy Military Medical University Affiliated Changhai Hospital, Shanghai 200433, China; ^3^Department of Pancreatic and Hepatobiliary Surgery, Cancer Hospital, Fudan University Shanghai Cancer Center, Shanghai 200032, China; ^4^Central Laboratory for Medical Research, Shanghai Tenth People's Hospital, Tongji University School of Medicine, Shanghai 200072, China

## Abstract

Non-small-cell lung cancer (NSCLC) is one of the most devastating diseases worldwide. The study is aimed at identifying reliable prognostic biomarkers and to improve understanding of cancer initiation and progression mechanisms. RNA-Seq data were downloaded from The Cancer Genome Atlas (TCGA) database. Subsequently, comprehensive bioinformatics analysis incorporating gene ontology (GO), Kyoto Encyclopedia of Genes and Genomes (KEGG), and the protein-protein interaction (PPI) network was conducted to identify differentially expressed genes (DEGs) closely associated with NSCLC. Eight hub genes were screened out using Molecular Complex Detection (MCODE) and cytoHubba. The prognostic and diagnostic values of the hub genes were further confirmed by survival analysis and receiver operating characteristic (ROC) curve analysis. Hub genes were validated by other datasets, such as the Oncomine, Human Protein Atlas, and cBioPortal databases. Ultimately, logistic regression analysis was conducted to evaluate the diagnostic potential of the two identified biomarkers. Screening removed 1,411 DEGs, including 1,362 upregulated and 49 downregulated genes. Pathway enrichment analysis of the DEGs examined the Ras signaling pathway, alcoholism, and other factors. Ultimately, eight prioritized genes (GNGT1, GNG4, NMU, GCG, TAC1, GAST, GCGR1, and NPSR1) were identified as hub genes. High hub gene expression was significantly associated with worse overall survival in patients with NSCLC. The ROC curves showed that these hub genes had diagnostic value. The mRNA expressions of GNGT1 and NMU were low in the Oncomine database. Their protein expressions and genetic alterations were also revealed. Finally, logistic regression analysis indicated that combining the two biomarkers substantially improved the ability to discriminate NSCLC. GNGT1 and NMU identified in the current study may empower further discovery of the molecular mechanisms underlying NSCLC's initiation and progression.

## 1. Introduction

As one of the most devastating diseases worldwide, lung cancer causes nearly 1.6 million mortalities each year [1–3]. Approximately 85% of lung cancers are characterized as non-small-cell lung cancer (NSCLC) [4–6], which is typically classified into two subtypes, squamous cell carcinoma (SCC) and adenocarcinoma (AD), using standard pathology methods [7–10]. Tobacco smoking is the most common risk factor for lung cancer. Smoking is also associated with multiple risks, including worse tolerance of treatment, higher risk of failure and second primary tumors, and poorer quality of life. Indeed, it has become clear that the significant reduction in tobacco consumption would result in the prevention of a large fraction of lung cancer cases and other smoking-related diseases [11–13].

In addition, other factors such as air pollution, poor diet, occupational exposure, and hereditary factors have been reported in association with NSCLC in nonsmokers [14–16]. Over the past few years, newly developed cytotoxic agents, including paclitaxel, gemcitabine, and vinorelbine, have emerged to offer multiple therapeutic choices for patients with LUAD [17–20]. However, chemotherapy for advanced NSCLC is often considered ineffective or excessively toxic [21–23].

In an attempt to improve treatments for NSCLC, new therapeutic strategies, such as the development of noncytotoxic targeted agents, have emerged [24–27]. Moreover, the targeted therapies have significantly improved clinical outcomes in a subset of lung cancer patients whose tumors harbor EGFR [28], ALK [29, 30], and HER2 alterations [31–33].

Despite recent advances in cancer treatment, unfortunately, the current five-year survival rate of NSCLC remains unsatisfactory [34–37]. Thus, it is imperative to identify potential biomarkers and explore NSCLC's underlying biological mechanisms.

In recent years, bioinformatics analysis has been utilized as a powerful tool to explore novel prognostic and therapeutic biomarkers and to unveil the potential mechanisms of NSCLC [38–41]. For instance, a novel model including seven genes was reported to indicate a promising prognostic biomarker for lung SCC patients using integrated bioinformatics methods [41–43]. In addition, studies used comprehensive bioinformatics analysis to show that the cell cycle pathway may play a significant role in NSCLC in nonsmokers [44–47].

In the present study, RNA-Seq data were downloaded from The Cancer Genome Atlas (TCGA) database. Then, the EdgeR package was applied to uncover differentially expressed genes (DEGs) between NSCLC tissues and normal tissues. Using the resulting data, this study is aimed at unveiling the underlying molecular mechanism of NSCLC onset and progression through gene ontology (GO), Kyoto Encyclopedia of Genes and Genomes (KEGG) pathway enrichment analysis, and the protein-protein interaction (PPI) network. Subsequently, cytoHubba, a novel Cytoscape plugin, was used to reveal the hub genes from 12 topological analysis methods. Furthermore, the prognostic and diagnostic values of the hub genes were further confirmed by survival analysis and receiver operating characteristic (ROC) curve analysis.

The screening revealed two key genes, GNGT1 and NMU, and the protein expressions of these genes were validated by the Human Protein Atlas online database at the system level. Their genetic alteration and coexpression were also revealed. Finally, a logistic regression model was built to evaluate the combined diagnostic capability of GNGT1 and NMU.

## 2. Materials and Methods

### 2.1. Downloading of TCGA Datasets and DEG Screening

The mRNA expression data of NSCLC patients were downloaded from the TCGA database (https://cancergenome.nih.gov/) [48]. The criteria used were as follows: primary site (lung), data category (Transcriptome Profiling), project ID (TCGA-LUAD and TCGA-LUSC), experimental strategy (RNA-Seq), and workflow type (HTSeq-counts). The other filters were kept as default. Practical Extraction and Reporting Language (Perl) was utilized to extract the sample information, generate the mRNA expression matrix, and annotate gene symbols. Finally, data from a cohort containing 1,145 samples were obtained from TCGA. Of these 1,145 samples, there were 108 normal tissue and 1,037 NSCLC samples, respectively. The EdgeR package from Bioconductor was used to screen the DEGs between normal tissue and NSCLC [49–51]. The adjusted *P* < 0.001, and fold change (FC) > 4 were set as the cutoff criteria.

### 2.2. DEG Functional Enrichment Analysis

Gene ontology (GO) analysis provides a standardized description of gene products in terms of molecular function (MF), biological process (BP), and cellular component (CC) [52]. The Kyoto Encyclopedia of Genes and Genomes (KEGG) is a database offering gene functional meanings and expressed proteins [53]. GO and KEGG enrichment analyses were conducted using the powerful online tool DAVID (DAVID, https://david.ncifcrf.gov/) and visualized by the R package “ggplot2” [54]. In addition, *P* < 0.05 was considered to indicate statistical significance.

### 2.3. Constructing the Protein-Protein Interaction Network

The Search Tool for the Retrieval of Interacting Genes (STRING, https://string-db.org/) database, a database that integrates all functional interactions between proteins, was used to build the PPI network [55]. An interaction score of ≥0.4 was considered statistically significant.

### 2.4. Hub Gene Selection and Analysis

A Cytoscape plugin, Molecular Complex Detection (MCODE), was utilized to screen modules of PPI networks with a node score cutoff of 0.2, degree cutoff of 2, *k*-core of 2, and max depth of 100. A *P* value of <0.05 was considered statistically significant. Next, the DEGs were ranked by cytoHubba [56], which contains 12 algorithms: Maximal Clique Centrality, Edge Percolated Component, Betweenness, Density of Maximum Neighborhood Component, Degree, Bottleneck, Eccentricity, Closeness, Radiability, Maximum Neighborhood Component, Stress, and Clustering Coefficient. The MCODE and cytoHubba results were combined to identify the hub genes.

### 2.5. Survival Analysis of Hub Genes

Whether the expression level of hub genes was associated with overall survival was investigated using the Kaplan–Meier plotter (http://www.kmplot.com/). An online database is capable of assessing the effect of 54,675 genes on survival using 10,461 cancer samples, including samples from 2,437 lung cancer, 1,065 gastric cancer, 1,816 ovarian cancer, and 5,143 breast cancer patients. *P* < 0.05 (Cox) was considered statistically significant.

### 2.6. ROC Curve

The ROC curve analysis was applied to evaluate the specificity and sensitivity of the hub genes. The area under the curve (AUC) and *P* value were calculated. *P* < 0.05 was considered to denote statistical significance.

### 2.7. Validation of Hub Genes

The expression level of hub genes in LUAD was validated by Oncomine (https://www.oncomine.org/resource/login.html) [57]. The threshold was set as the following: *P* < 1E − 4, fold change > 2, and gene ranking in the top 10%.

### 2.8. Human Protein Atlas

The Human Protein Atlas (https://www.proteinatlas.org) is an online website that includes immunohistochemical data of nearly 20 types of tumors [58]. In our study, immunohistochemical images were used to directly compare the expression of biomarkers in normal and NSCLC tissues. The intensity of antibody staining indicated the protein expression of hub genes.

### 2.9. Genetic Alteration of Hub Genes

The cBio Cancer Genomics Portal (http://www.cbioportal.org/) is an open platform that provides visualization, analysis, and downloads of large-scale cancer genomic datasets for various cancer types [59]. Complex cancer genomic profiles can be easily obtained using the portal's query interface, enabling researchers to explore and compare genetic alterations across samples. cBioPortal was used to explore genetic alterations, coexpression, and overall survival of two hub genes, GNGT1 and NMU.

### 2.10. Statistical Analysis

SPSS version 23.0 (SPSS Inc., Chicago, IL, USA) was used to perform logistic regression analysis. ROC curves were generated to evaluate the diagnostic accuracy of GNGT1 and NMU, and AUC was used to evaluate sensitivity and specificity.

## 3. Results

### 3.1. Identification of DEGs in NSCLC

**Figure 1:**
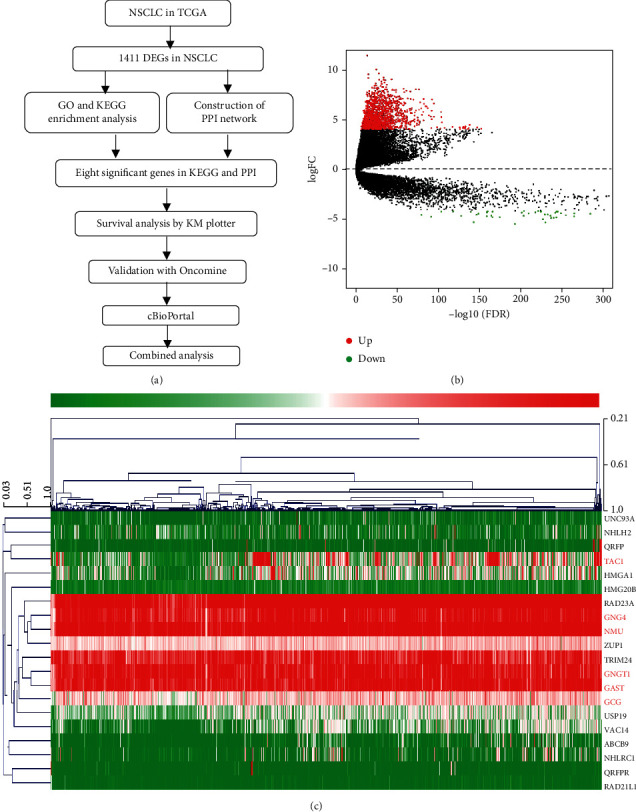
Identification of DEGs in NSCLC. **(**a) Workflow for the identification of key pathways and genes between non-small-cell lung cancer and normal samples. (b) DEGs between LUAD tissue and normal tissue. The volcano plot showed 1,411 DEGs. The red dots represented the upregulated genes, while the green dots represented downregulated genes. DEGs: differentially expressed genes. (c) Heatmap of the 20 upregulated and downregulated DEGs. The red color represents high expression, and the blue color represents low expression.

The workflow is shown in Figure 1(a). DEGs were identified using the criteria of *P* < 0.001 and FC > 4. A total of 1,411 DEGs were screened out between NSCLC and normal samples, including 1,362 upregulated genes and 49 downregulated genes (Figures 1(b) and 1(c)).

### 3.2. Functional and Pathway Analysis of DEGs

To further investigate the specific function of these genes, all DEGs were uploaded to the online tool DAVID. GO analysis revealed that in terms of BP, the DEGs were associated with nucleosome assembly, transcription from RNA polymerase II promoter, telomere organization, flavonoid glucuronidation, and DNA replication-dependent nucleosome assembly.

**Figure 2:**
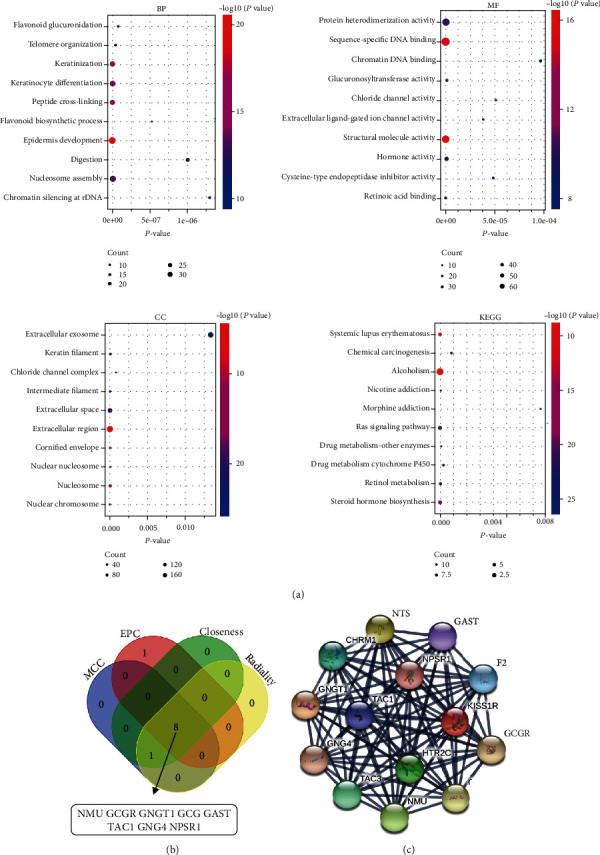
Functional and pathway analysis of DEGs. (a) GO and KEGG analysis of DEGs. *P* value is displayed on the *x*-axis, and GO function enrichment and KEGG pathway are shown on the *y*-axis. GO: gene ontology. KEGG: Kyoto Encyclopedia of Genes and Genomes. (b) The overlapping genes of different algorithms selected by cytoHubba. (c) The most significant modules obtained from the PPI network. PPI: protein-protein interaction.

When examined in terms of MF, DEGs were enriched in protein heterodimerization activity, retinoic acid-binding, hormone activity, glucuronosyltransferase activity, and extracellular ligand-gated ion channel activity. Regarding CC, the DEGs were mainly enriched in the extracellular region, cornified envelope, nucleosome, extracellular space, and intermediate filament. KEGG analysis found that the DEGs were predominantly involved in the Ras signaling pathway, nicotine addiction, steroid hormone biosynthesis, alcoholism, and systemic lupus erythematosus (Figure 2(a)).

### 3.3. PPI Network Construction, Module Analysis, and Hub Gene Selection

The PPI network was constructed using the STRING database and visualized in Cytoscape. The PPI network consisted of 787 nodes and 2,104 edges, including 1,362 upregulated genes and 49 downregulated genes. The overlapping genes of different algorithms selected by cytoHubba were GNGT1, GNG4, NMU, GCG, TAC1, GAST, NPSR1, and GCGR (Figure 2(b)). The top modules were then extracted from the PPI network (Figure 2(c)).

### 3.4. Survival Analysis

**Figure 3:**
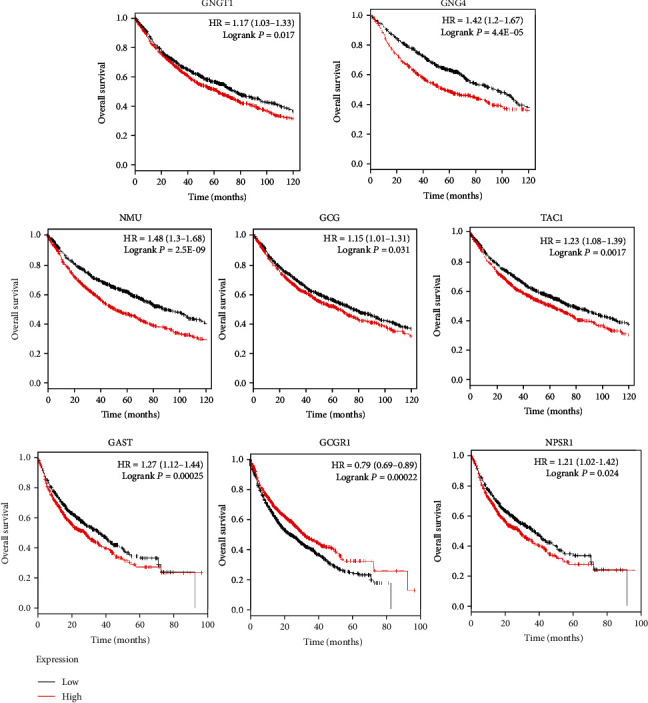
The prognostic value of hub genes in NSCLC patients. Kaplan–Meier curve analysis between hub gene expression and prognosis in NSCLC patients from the KM plotter database.

The Kaplan–Meier plotter was used to predict the prognostic value of the six identified hub genes. The results demonstrated that high expressions of GNGT1 (HR = 1.17 (1.03–1.33), logrank *P* = 0.017), GNG4 (HR = 1.42 (1.2–1.67), logrank *P* = 4.4e − 05), NMU (HR = 1.48 (1.3–1.68), logrank *P* = 2.5e − 09), GCG (HR = 1.15 (1.01–1.31), logrank *P* = 0.031), TAC1 (HR = 1.23 (1.08–1.39), logrank *P* = 0.0017), GAST (HR = 1.27 (1.12–1.44), logrank *P* = 0.00025), GCGR (HR = 0.79 (0.69–0.89), logrank *P* = 0.00022), and NPSR1 (HR = 1.21 (1.02–1.42), logrank *P* = 0.024) were associated with worse overall survival for NSCLC patients (Figure 3).

### 3.5. ROC Curve

**Figure 4:**
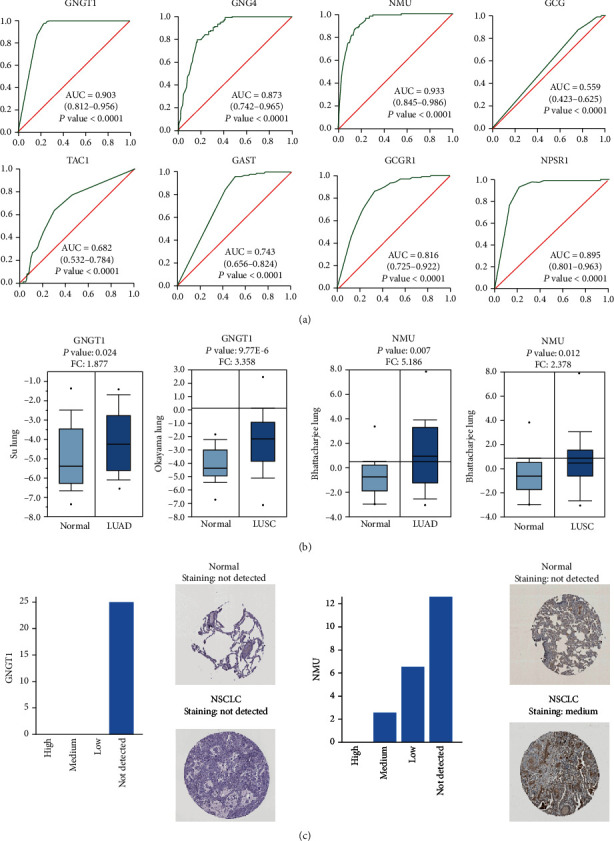
The expression and prognostic value of four hub genes in NSCLC patients. (a) The ROC curves of hub genes. AUC and *P* values of each hub gene are displayed in the plot. ROC: receiver operating characteristic. AUC: area under the curve. (b) Expression levels of significant genes compared between different types of NSCLC and normal tissues from the Oncomine platform. Fold changes and *P* values of each hub gene are displayed in the plot. (c) Immunohistochemical analysis of GNGT1 and NMU in normal tissues and NSCLC tissues from the Human Protein Atlas.

According to ROC curve analysis, the AUCs of GNGT1, GNG4, NMU, GCG, TAC1, GAST, GCGR1, and NPSR1 were 0.9027 (*P* < 0.0001), 0.8729 (*P* < 0.0001), 0.9323 (*P* < 0.0001), 0.559 (*P* < 0.0432), 0.6822 (*P* < 0.0001), 0.7426 (*P* < 0.0001), 0.816 (*P* < 0.0001), and NPSR1 0.8949 (*P* < 0.0001), respectively (Figure 4(a)).

### 3.6. Validating Hub Gene Expression

The Oncomine database was used to validate the expression of hub genes. The results demonstrated that GNGT1 had high expression in LUAD (*P*: 0.024, FC: 1.877) and LUSC (*P*: 9.77E-6, FC: 3.358). In Bhattacharjee's study, NMU showed high expression in LUAD (*P*: 0.007, FC: 5.186) and LUSC (*P*: 0.012, FC: 2.378) (Figure 4(b)).

### 3.7. Human Protein Atlas

After studying the mRNA expression of hub genes in NSCLC, we tried to explore the protein expression of hub genes using the Human Protein Atlas. The results revealed that NMU protein was not expressed in normal lung tissues, whereas medium expression of NMU protein was observed in the NSCLC tissues. However, GNGT1 was not detected in either normal lung tissues or NSCLC tissues (Figure 4(c)).

### 3.8. Genetic Alteration of Hub Genes

**Figure 5:**
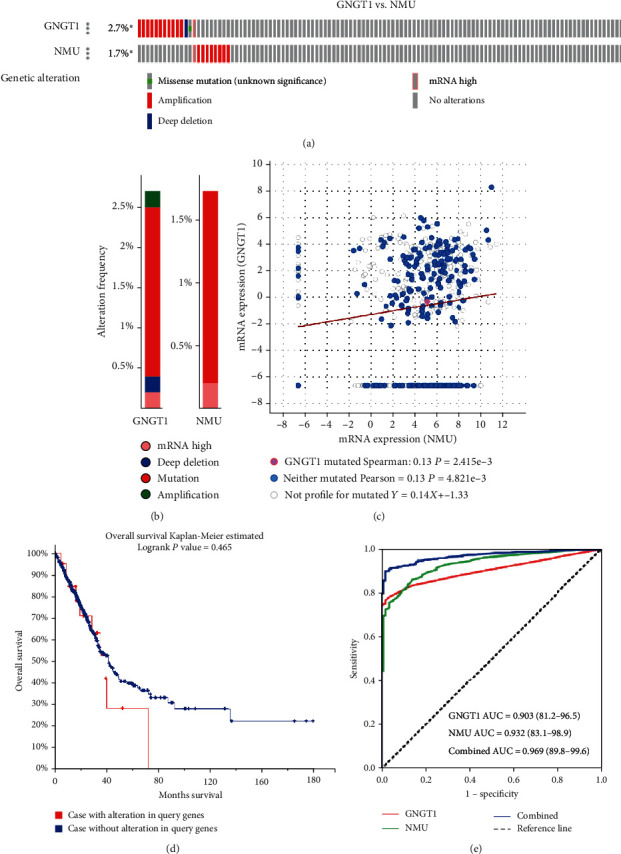
The expression and prognostic value of GNGT1 and NMU in NSCLC patients. (a) Genetic alteration of GNGT1 and NMU genes in NSCLC patients. (b) Illustration of the alteration frequency of GNGT1 and NMU genes in NSCLC patients. (c) Coexpression between GNGT1 and NMU. (d) Overall survival analysis for GNGT1 and NMU genes in NSCLC patients. (e) Combined diagnosis of GNGT1 and NMU genes in NSCLC patients.

The two hub genes altered in 22 (4%) of the 584 patients, and the frequency of alteration of each hub gene, are shown in Figure 5(a). GNGT1 and NMU were altered most often (2.7% and 1.7%, respectively), with mutation, amplification, and mRNA upregulation as the main types of alterations observed (Figure 5(b)). The expression of GNGTA was correlated with NMU (Spearman: 0.13, *P* = 2.415e − 3; Pearson = 0.13, *P* = 4.821e − 3) (Figure 5(c)). Patients with CYP1A2 and GSTA3 alteration had worse overall survival than patients without CYP1A2 and GSTA3 alteration (*P* = 0.465) (Figure 5(d)).

Notably, according to the ROC curve analysis, the AUC of GNGT1 was 0.903 (*P* < 0.0001). For NMU, the AUC was 0.932 (*P* < 0.0001). The AUC was largest when GNGT1 was combined with NMU (AUC = 0.969, *P* < 0.0001) (Figure 5(e)).

## 4. Discussion

Elucidating the molecular mechanisms of the initiation and development of NSCLC would benefit the early diagnosis and targeted therapy efforts [60–63]. In this study, we identified 1,362 upregulated genes and 49 downregulated genes and selected GNGT1, GNG4, NMU, GCG, TAC1, GAST NPSR1, and GCGR as hub genes using Molecular Complex Detection (MCODE) and cytoHubba. These genes were primarily enriched in terms of the Ras signaling pathway, steroid hormone biosynthesis, nicotine addiction, alcoholism, steroid hormone biosynthesis, and systemic lupus erythematosus.

The Ras signaling pathway is closely related to the occurrence and progression of most human tumors [64–67]. The activation of RAS-RAF-MEK-MAPK in gene transcription regulation can promote proliferation, migration, and angiogenesis of cancer cells [68–70]. RAS-PI3K interaction is an important signaling node and potential therapeutic target in EGFR-mutant lung cancer [71–73]. In addition, steroid hormones were not previously considered to be involved with lung function [74–76]. However, numerous studies have reported that steroid hormones are important in normal lung development and function [77], as well as in the pathogenesis of pulmonary diseases, including lung cancer [78–81].

Cigarette smoking is a well-known risk factor for the occurrence and progression of malignant diseases [82–85]. Nicotine, the major constituent in cigarette smoke, plays key roles in cancer progression [86–89]. Nicotine likely promotes lung cancer cell proliferation by upregulating HIF-1*α* and SOCC components [90–93]. It was demonstrated that nicotine increased NSCLC cell proliferation through nicotinic acetylcholine receptor-mediated signals [94–97]. Nicotine can also induce the expression of embryonic stem cell factor Sox2, which is indispensable for self-renewal and the maintenance of stem cell properties in NSCLC cells [98–100].

Several studies have been conducted to investigate the association between alcohol and lung cancer. Some studies have reported that alcohol is linked to a number of human diseases, including cancers [101–103]. Interestingly, another report shows that alcohol has nothing to do with lung cancer [104]. Thus, conducting further experiments is necessary to confirm whether lung cancer is attributable to alcohol abuse. All in all, the findings of these studies are consistent with our results.

In the current study, the expressions of GNGT1 and NMU were low both in the Oncomine and TCGA databases, indicating that GNGT1 and NMU may play a role as oncogenes. The transducin *γ*-subunit gene (GNGT1) has been localized to human chromosome 7 [104] and is associated with various forms of cancer [105–108]. GNGT1 exerts effects in different tissues regulating cell proliferation, migration, adhesion, and apoptosis [109–111]. One study showed that GNGT1 could serve as a marker of medulloblastoma [112]. GNGT1 can be utilized to differentiate gastrointestinal stromal tumor and leiomyosarcoma, two cancers that have very similar histopathology, but require very different treatments [113–115]. In the current study, GNGT1 was significantly upregulated and high mRNA expression of GNGT1 was associated with poor overall survival in NSCLC patients. Furthermore, KEGG analysis showed that GNGT1 was involved in the Ras signaling pathway. Therefore, it is reasonable to regard GNGT1 as a hub gene of NSCLC. Further studies are needed to better understand GNGT1's association with NSCLC.

Neuromedin U (NMU) has been reported to exhibit early alterations associated with cancer, including lung cancer, pancreatic cancer, breast cancer, renal cancer, and endometrioid endometrial carcinoma, through promoting migration, invasion, glycolysis, a mesenchymal phenotype, a stem cell phenotype of cancer cells, and resistance to the antitumor immune response [116–118]. It is overexpressed in pancreatic cancer and increases the cancer invasiveness through the hepatocyte growth factor c-Met pathway [119–121]. A role has also been implicated for NMU in human breast cancer and endometrial cancer [122–124]. The protein encoded by NMU can amplify ILC2 to drive allergic lung inflammation [125]. NMU is regulated by RhoGDI2, a metastasis inhibitor, which can be used as a target for lung metastasis. The expression of NMU is negatively correlated with prognosis in most types of cancer [126–128]. In the present study, the higher mRNA and protein expression of NMU were negatively correlated with overall survival. Therefore, our results are in line with these previous studies, which indicated that NMU may be directly or indirectly important in NSCLC development.

Moreover, to explore the predictive ability of GNGT1 and NMU, logistic regression analysis was performed. The logistic regression analysis showed a probabilistic nonlinear regression, which has functions in discrimination and prediction. Notably, according to logistic regression analysis, the AUC of the ROC curve of GNGT1 was 0.903 (*P* < 0.0001), and the AUC of NMU was 0.932 (*P* < 0.0001). Combining the two biomarkers enabled a relatively high capacity for discrimination between NSCLC and normal patients, with an AUC of 0.969, indicating that the combined test of GNGT1 combined with NMU was superior to testing for either gene individually, with better clinical accuracy and higher diagnostic value. Therefore, it is of high scientific value to use a logistic regression model as a diagnostic model for NSCLC.

In conclusion, our results identified two hub genes, GNGT1 and NMU, as prognostic target genes, and highlighted their probable role in NSCLC. Nevertheless, a few limitations to this study should be acknowledged. Because all the data analyzed in the current study were retrieved from the online databases, further independent experiments are required to validate our findings and to explore the molecular mechanism of the hub genes in NSCLC development and progression.

## Data Availability

All data generated or analyzed during this study are included in this article.

## Additional Points

*Impact Statement.* GNGT1 and NMU identified in the current study may empower further discovery of the molecular mechanisms underlying NSCLC's initiation and progression.
